# Participation and support – associations with Swedish pupils’ positive health

**DOI:** 10.1080/22423982.2017.1373579

**Published:** 2017-09-14

**Authors:** Maria Warne, Kristen Snyder, Katja Gillander Gådin

**Affiliations:** ^a^ Department of Health Sciences, Mid Sweden University, Östersund, Sweden; ^b^ Department of Health Sciences, Mid Sweden University, Sundsvall, Sweden

**Keywords:** Adolescents, health scale, health promotion, participation, school, teacher support

## Abstract

From the perspective of salutogenesis, schools have opportunities to create supportive environments for health and well-being, but there is a need for more knowledge about positive health determinants in the school setting. The aim of this study was to analyse adolescents’ self-reported positive health and its association with supportive factors in the school environment. Data was derived from a cross-sectional study in which pupils were aged 12–16 (n=1527). A positive health scale was used to examine the association of positive health with the following determinants: classroom participation; teacher support; peer support; parental support; and personal relative affluence. Data was analysed with multiple logistic regression. The results showed that positive health was associated with classroom participation and support from teachers and parents more commonly among boys than girls. All determinants were significantly associated with pupils’ positive health. The conclusion is that students’ positive health is strongly associated with support from the school. Classroom participation and support are major concerns for the health of pupils, and it is essential to develop these aspects of the school environment.

## Introduction

Adolescents’ access to education is one of the strongest determinants of health all over the world, together with access to secure and supportive families, peers and schools [1]. Most of the research examining determinants of health still focuses on what causes illness and diseases and little is directed towards what leads to positive health [2]. Seligman’s [3] work on the subjective dimension of health, defined as a positive and subjective experience of the individual characterised by a sense of energy, vitality, determination, and optimism, has given rise to the notion of positive health. Researchers have discussed whether the perspective taken on health matters and whether health and illness are different constructs or variations on a theme [4]. Despite these different standpoints, it has been argued that there is a need for more knowledge on environmental factors that promote young people’s positive health [5]. Adolescence is a crucial time in young people’s lives and conditions for different generations are changing [6]. Given the view that health is determined by structural and environmental factors [1], a knowledge of structural health-protecting and health-promoting determinants is required [7]. The focus on health promotion, on resources, and on strengthening health is in line with the salutogenic theory, although Antonovsky [8] focused more on what leads to health, rather than on defining the concept of health. He analysed why people in stressful situations still had good health, or even improved their health, despite their vulnerability. Inspired by this salutary paradigm, in the present study the focus is on factors in the school environment that could be useful in relation to the promotion of positive health in schools.

Health promoting schools (HPS), a framework created by the World Health Organisation in the mid-1980s, has been suggested as a way of increasing the health of children and adolescents [9]. The development of HPS was shaped by the health sector, with the purpose of facilitating health gains [10]. Even if the health effects of using this framework are small, this is still considered important by Langford *et al*. [11] in their systematic review and meta-analysis. However, the development of health promoting schools has been slow and one of the explanations for this is that health is not the main concern of schools [12]. Instead, health and education are seen to be unintegrated parallel processes in the core business of schooling [12,13]. Another contributing factor to the slow development of HPS could be that most health interventions at school, along with research projects on health, are pathogenic in their focus on what causes ill health; they are less concerned with what leads to and promotes positive health [2]. Rowling [13] argues that there is need for a whole school approach in which well-being and school achievement are seen to be mutually dependent. In order to consider HPS as a trustworthy and helpful framework to increase the health and learning of children and adolescents, it is necessary to know more about the environmental factors that promote young people’s health [5].

In a qualitative study [14], students in upper secondary school discussed their view of factors that promote health and learning and they found that genuine participation and influence in the classroom, through the practice of aspects of democracy, were important. The students also identified teacher-, parental- and peer-support as health-promoting factors [14]. Beginning with participation, Griebler *et al*. [15] conclude in their review that participation can have positive effects on, for example, satisfaction, motivation, self-esteem, and self-efficacy. Ahlström [16,17] measured students’ participation and influence by asking questions related to the practice of democracy, such as, for example, standing up for one’s opinion, respecting each other’s opinions, and being able to review facts critically. He also included questions on communication, about, for example, listening to others’ opinions, and expressing one’s own opinions in such a way that others listen. Co-operation was another focus of his questions and he was interested in measuring one’s awareness of the associations between actions and consequences. Åkerström [18] asked young people at school about their perspective on participation and found that they also mentioned communication as an important aspect of participation, together with social, educational and decision-making factors. The definition of participation and the operationalization of the concept developed and used by Ahlström [16,17] is interesting, since he found that a high level of participation in the classroom was associated with high school achievement and a low level of bullying. Carlerby *et al*. [19] measured participation in line with Ahlström and found an association between low classroom participation and psychosomatic symptoms.

The relationship between low support from parents, peers and teachers on the one hand and psychosomatic health complaints on the other has been commonly reported [1]. It has also been reported that low support during adolescence also increases the level of functional somatic symptoms during the life course [20]. However, the positive relation between social support and health is more rarely measured, although it has been done by, for example, Danielsen *et al*. [21], who examined social support in relation to life satisfaction.

The relationship between socioeconomic status (SES) and health problems is well documented and, for example, the study “Health Behaviour in School-aged Children” shows that socioeconomic inequality has increased in relation to health, e.g. life satisfaction [22]. There are different ways to operationalise SES in studies of school children (e.g. parents’ occupation; education; family affluence; and personal affluence), but Elgar *et al*. [23] show that relative deprivation related more to psychosomatic symptoms among adolescents than did absolute deprivation.

Despite the focus of participation in health promotion projects, quantitative studies that have operationalised the concept and showed associations to positive health are lacking. Since most studies on adolescents’ health focus on problems of ill health and risk factors in the environment, there is a need to increase our understanding of supportive environments and of the significance of an advantaged socioeconomic position.

The objective of this study was to increase the knowledge about health-promoting factors related to the school environment by using a positive health scale for that purpose. The specific question has to do with whether participation, social support and personal affluence are associated with positive health.

## Methods

This study draws on data from the Youth Health Development project, conducted between 2009 and 2012 in a medium-sized municipality, with ~59,000 inhabitants in the north of Sweden. The overall aim of the project was to increase the understanding of factors related to mental health among youth and to develop methods for school health promotion. The data in this study comes from a questionnaire distributed in January 2011. Compared to people in Sweden in general more of the inhabitants in this area live in a detached house or town house and fewer inhabitants have a foreign background compared with the Swedish average.

### Participants and procedure

All public and independent schools with junior high school students (aged 12–16 years in grades 6–9) were invited to participate in the study; one of the four independent schools and all nine public schools accepted. An informative letter was sent to the students and their parents. Participation in the study was voluntary and parents were informed that they could actively decline to give permission for their children’s participation. The project was approved by the Umeå Human Research Ethics Board (Dnr: 09-179M). In total, 1,527 students (52.3% girls, 47.7% boys) answered the questionnaire. The response rate was 80%. The questionnaire was provided to the students via their school e-mail with a unique password and an opportunity to decline participation. The questionnaire was completed during school hours in a computer room. All schools were given extra resources so that at least one member of staff could be present to make sure that all pupils were able to complete the questionnaire in private and did not disturb one other.

### Measures

*Positive health* was measured as a dependent variable using the *Positive Health Scale* (PHS), which was validated by Warne *et al*. [24]. Inspired by the Ottawa Charters [25] definition of health, the PHS scale was designed based on the Salutogenic theory to identify resources that can be used to strengthen an individual and improve health. The PHS measures mental and cognitive aspects of health using positive worded questions [24].

Nine items are included in the scale all starting with “In the last 6 months, how often have you ...” and continued with one of the following: *been alert*; *happy*; *relaxed*; *creative*; *decisive*; *found it easy to concentrate*; *felt well*; *had energy*; and *been social*. The response alternatives were: always; often; sometimes; rarely; and never. The index runs from 0 to 36, measuring a higher number as indicative of more positive health. The scale showed significant results when it was tested for normality by the Kolgomorov-Smirnov statistics. Because of the non-normality distribution, the scale was dichotomised by dividing the responses into tertiles, in line with Bosma *et al*. [26]. The highest tertile indicated high positive health and the cut-off point was placed on ≥27. The internal reliability, determined by Cronbach’s Alpha, was 0.89.

*Classroom participation* was measured in three dimensions of participation by the *Social and Civic Objectives Scale* (SCOS) [16,17]. The internal reliability was 0.92. The scale is built on the following dimensions: (1) *Democratic competence*: (a) standing up for one’s opinion, (b) respecting each other’s opinions, and (c) being able to review facts critically (score 3–18); (2) *Communication*: (a) listening to others’ opinions, (b) being good at explaining what and how one thinks, (c) expressing one’s own opinions in such a way that others listen, and (d) participating in most of the discussions during the classes (score 4–24); and (3) *Co-operation*: (a) being aware of the associations between actions and consequences, (b) being able to resolve conflicts in the class, (c) being good at co-operating, and (d) valuing/appreciating the opportunity to co-operate (score 4–24). Examples of questions: Do you think that your classmates respect each other’s opinions? Do you think your classmates listen to each other? Are your classmates good at cooperation? Each question had six answer options, from “yes absolutely”; to “no, not at all”; and “I do not know”. In line with Carlerby *et al*. [19], the single score of each component was categorised to tertiles and coded low, middle and high: *Democratic competence* 3–5, 6–9 and 10–18, *Communication* 4–7, 8–11 and 12–24, and *Cooperation* 4–7, 8–12 and 13–24. Those individuals who were allocated to the low and middle tertile in all three components were used as referents and coded 0, the third tertile coded 1.

*Teacher support* was measured by an index of five items scored 0–20 (Cronbach’s Alpha = 0.87). The teacher support scale was derived from earlier studies [27,28]. Five questions were chosen that reflected different aspects of support from teachers. The questions were then tested and validated as a scale for teacher support by a principal component analysis (PCA) showing a one-way solution. The questions were: Do you think teachers will give you help when you need it?; Do you think your teachers would notice if you did not carry on with your school work?; Do you think your teachers treat you fairly?; Do your teachers praise and encourage you?; and Are there any adults at school you can talk with if you have any problems?. The answer alternatives were: never; seldom; sometimes; often; and always. The scale was dichotomised at the upper tertile, based on the distribution of the answerers coded as 1, indicating a high degree of support.

*Peer support* was measured by an index of five items scored 0–20 (Cronbach’s Alpha = 0.71). Does it happen that you are alone even if you do not want to be? Do you have as many friends as you want to have? Are you called rude words at school? Do you feel excluded from the peer group? Are you afraid of other pupils at school? The answer alternatives were never; seldom; sometimes; often; and always. The scale was dichotomised, based at the distribution of the answers, at the upper tertile, coded as 1, indicating a high degree of support.

*Parental support* was measured by the item: Do you usually talk about most things with your mother and/or father? The possible answers were never; seldom; sometimes; often; always; and do not have any or never met them. A high degree of parental support was defined as the students always or often talking about most things with both their mother and their father [2]. Medium parental support was defined as talking always or often either with their father or their mother [1], while other answers (sometimes; seldom; never; or do not have father/mother) were defined as low parental support, with the latter used as referent.

*Personal relative affluence* was used as a proxy for SES and measured by the item: If you consider the past 3 months, have you had enough money to be able to do the same things as your friends? The response choices were never; seldom; sometimes; often; and always. The answer alternatives: “always” and “often” were categorised as having high personal affluence, while the response alternatives: “sometimes”, “rarely” and “never” had low personal relative affluence. It is important to note that absolute SES of a student is not determined by this variable; however, it is shown to be a stable indicator of relative economic affluence, which has been correlated in another study to mental health [25].

### Statistical analyses

Comparisons between girls and boys were conducted with chi-squared analysis. A *p*-value less than 0.05 was accepted as statistically significant. To test the internal reliability of the scales, Cronbach’s Alpha was used. For construction of index for teacher support, a principal component analysis was made. Spearman’s rho was used to test for multicollinearity. All correlations were below r=0.30, which was judged as satisfactory for inclusion in the model. To analyse the association between positive health and the predictors, a logistic regression analysis was used to estimate the crude positive odds ratio (POR). All variables that were significant in the crude analysis for boys or girls were included in the multivariate regression analysis. A confidence interval of 95% was used for the regression analysis.

## Results

Table 1 shows that significantly more boys than girls reported positive health. Boys also had a more positive situation in comparison with the girls regarding participation in the classroom, teacher support, and parental support. About four out of five pupils reported that they always or often had had as much money as their friends during the last 3 months.

**Table 1.** Prevalence of each variable for boys and girls (%). P-value from Chi-square test.

Outcome variable	Boys	Girls	p-value gender
PHS			
Yes 1 = 27–36	39.7	24.0	
No 0 = 0–26	60.3	76.0	< 0.001
*Determinants*			
Participation			
Low	64.4	72.9	
High	35.6	27.1	< 0.001
Teacher support			
Low	62.2	72.2	
High	37.8	27.8	0.000
Peer support			
Low	67.0	71.7	
High	33.0	28.3	0.057
Parental support			
No	30.8	29.9	
Mother or father	26.1	33.0	0.017
Mother and father	43.0	37.1	
Personal relative affluence			
HIgh	80.8	80.3	
Low	19.2	19.7	0.816

PHS, Positive Health Scale.

Table 2 shows that all determinants in the crude analysis were significantly related to positive health. However, parental support was only positively correlated when both parents were involved. In the adjusted model, all significant associations between the independent variables and positive health remained. A high level of participation in the classroom increased the probability of a high degree of positive health by nearly three times for both sexes. Girls and boys who reported high peer support were almost twice as likely to report a high level of positive health, in comparison with those who reported low and middle participation. Support from both parents increased the probability of a high level of positive health, whereas there was no statistical association between support from a single parent and a high level of positive health .

**Table 2.** Logistic regression analysis showing positive odds ratio (POR) with 95% confidence intervals (CI) for girls’ and boys’ positive health associated with the determinants. PHS ≥ 27 was determined as a high level of positive health.

			Crude POR				Adjusted POR			
	Proportion of boys and girls with positive health presented as a percentage within the determinants		Girls		Boys		Girls		Boys	
Determinants	Girls, % (n)PHS ≥ 27	Boys, % (n)PHS ≥ 27	POR	CI	POR	CI	POR	CI	POR	CI
Participation										
Low	17.4 (88)	26.9 (104)	1		1		1		1	
High	42.8 (80)	60.4 (128)	3.55	2.45–5.14	4.13	2.90–5.90	2.26	1.48–3.45	2.99	1.97–4.55
Teacher support										
Low	18.5 (93)	29.9 (106)	1		1		1		1	
High	37.9 (74)	59.3 (128)	2.70	1.88–3.90	3.42	2.40–4.87	1.65	1.06–2.56	2.28	1.49–3.50
Peer support										
Low	16.3 (84)	30.9 (124)	1		1		1		1	
High	41.4 (84)	56.5 (113)	3.62	2.52–5.21	2.90	2.04–4.12	2.57	1.69–3.90	2.29	1.49–3.53
Parent support										
No	16.1 (35)	29.5 (69)	1		1		1		1	
Mother or father	23.1 (58)	32.4 (34)	1.57	0.99–2.50	1.15	0.70–1.88	1.39	0.83–2.35	0.90	0.51–1.61
Both mother and father	34.6 (73)	54.7 (128)	2.77	1.75–4.38	2.89	1.97–4.23	1.81	1.06–3.10	1.72	1.07–2.76
Personal relative affluence										
No	10.8 (16)	24.6 (30)	1		1		1		1	
Yes	27.1 (163)	43.9 (234)	3.07	1.77–5.32	2.32	1.48–3.62	2.17	1.15–4.14	2.26	1.28–3.98
Total *R*^2^							0.20		0.28	

The multivariate model explained more of the variance in health for boys than girls. Nagelkerke R-square was 0.28 for boys, compared to 0.20 for girls. The value of the Hosmer- Lemeshow Goodness of Fit Test supports the model. The chi-square value was, for boys 5.736, with a significant level of 0.68; and for girls 6.499, with a significant level of 0.48.

## Discussion

This study showed that high participation in the classroom, support from teachers, peers and both parents, as well as high personal affluence increased the pupils’ odds of being in the group that reported high positive health. The importance of supportive families, schools and peers has been well described [1,29,30], but rarely in relation to positive health. The results will first discuss students’ health in relation to participation in the classroom and then relate this to support from teachers, pupils and parents and then in relation to personal affluence. Finally, there are some reflections on differences between girls and boys.

The association between pupils reporting high participation in the classroom and positive health has not been well described, although participation in relation to health problems such as bullying [17] and psychosomatic health complaints [19] has been studied. Our results show that experiencing participation in the classroom could also be a significant health factor for pupils; this makes it even more important for schools to work towards a high degree of communication, collaboration and democratic competence in line with Ahlström´s [16] measurements. Ahlström [17] also found that schools with a high level of participation increased students’ school achievement, which, in turn, could affect health positively. Based on the UN convention of the rights of the child [31], children have the right to be included in decisions that concern them and they have the right to speak and be heard.

Participation in terms of collaboration and communication is closely connected to inclusion. In a Swedish longitudinal study [32], being a member of a peer group during childhood was found to be significantly related to health in adult life. Other studies have described negative health factors in adolescents who lack peer support [33]. The relationship between feeling included and the social gradient is also a main concern for equity in health and schools should explore different kinds of pedagogical methods, such as critical pedagogy, for example [34], to increase pupils’ participation.

The association between high teacher support and pupils’ health, described by Danielsen *et al*. [21], was in line with this study, even though they used life satisfaction as an outcome variable. Teachers seem to be significant for school satisfaction in pupils [35], so how they connect to pupils is crucial.

The importance of parental support for children and adolescent health has been reported previously by, for instance, Resnick *et al*. [36]. The importance of good communication with parents for children’s well-being, including after a parental split-up, has been reported [37]. The importance of the family has been indicated in a study about social capital and children’s health, along with the effect of the school’s social capital on the children’s social well-being and on their subjective health complaints [38]. The results of this study on teachers’ support and participatory aspects related to a classroom situation also showed that supportive structures in the school organisation, along with good peer relationships, were strongly associated with the positive health of pupils. It is clear, therefore, that schools have an important role to play in children’s health, particularly if parental support is inadequate.

This study also showed that sex difference is not only a pattern in mental illness, but is also part of the pattern of mental health measured as positive health. Boys’ reports of a higher level of positive health are similar to other measurements of well-being as outcome, as is the case, for example, in Kidscreen [39] and Young HUNT II [40]. Differences between the sexes have also been frequently described when health problems are measured; girls score higher than boys in, for example, depressive symptoms and those related to other mental ill health issues [41]. From the results concerning the prevalence of determinants, we observed differences related to sex. Boys reported a higher degree of participation in the classroom and greater teacher support, in contrast to the findings of Bokhorst *et al*. [42], who found that girls had more teacher support than boys. One explanation of why the present study shows a contradictory result might have to do with the way the variable was measured. When teacher support was divided into emotional and instrumental support, as in a Swedish study [43], it was found that girls reported higher emotional support from teachers than boys, but boys reported more instrumental and appraisal support from teachers than girls did. Gendered structural differences have been established, for example, in relation to girls having fewer opportunities to make their voices heard [44], but more work needs to be done on sex- and gender-related differences in health in the school environment.

### Methodological considerations

This is the first study using the PHS with social and cognitive dimensions as a measurement of the positive aspects of mental health in adolescents. The intention was never to try to comprehend all the dimensions in the school environment that could probably be related to health. Rather, following Warne *et al*. [14] variables such as social support and classroom participation were selected based on the school as a supportive environment for health.

Since the study is cross-sectional, the results cannot establish any causal links between the variables. It is possible that the relationships go in both directions, but it is likely that a high degree of participation and support, along with good socioeconomic status, leads to a high degree of positive health to a greater extent than the other way around.

The main strength of this study was the relatively large sample size of nearly 1500 pupils aged between 12 and 16 years, and a high response rate. The use of valid scales was seen as a strength. The scales used in this study, such as PHS [24] and SCOS [17], have been shown to have high validity and reliability. The indices for measuring teacher support and peer support showed high internal consistency, as suggested by, for example, Hair *et al*. [45].

The data was self-reported by the adolescents. Measuring children’s and adolescents’ health this way has been found to be both valid and reliable [46]. No analysis was made of the non-response rate and in this group low-attending pupils with a lower level of health are probably more numerous than those who answered the questionnaire. This means that the pupils’ high degree of positive health could be over-estimated. The results might be generalisable to similar groups regarding age, cultural context, time and living conditions. The findings and conclusions should be interpreted taking these limitations into consideration.

### Implications for practitioners

The results of this study indicate clearly the importance of social support from both teachers and peers and of the supportive aspects of democratic practice, communication and co-operation in the classroom, including participation, that have to be taken seriously by the school organisation. This is also in line with the intentions of the Ottawa Charter regarding how different settings, e.g. schools, could enable health by creating supportive environments [47].

The school setting is a unique arena in which nearly 100% of all adolescents spend most of their days. Schools have opportunities to enable participation through a pedagogy that stimulates collaborative activities, civic education and dialogues and discussion through which pupils learn to listen to and respect each other [34]. The importance of strengthening social relations among pupils has to do with developing opportunities that involve both health and learning. Ahlström [17] found that student participation seems to have beneficial effects on students’ academic and social development and that a high level of participation is related to a lower level of bullying. If schools are to develop in this direction it is important that pupils’ opportunities to co-operate and to build social networks with strong ties increase.

In this article the focus is on factors that are associated with adolescents’ positive health. The study is in line with the work on how social determinants can improve the conditions of daily life for adolescents for whom education is the strongest structural factor for health [1]. With more knowledge about promoting factors, some of which are discussed in this article, we could reduce health inequalities and could better understand how to enable health at school. Viner *et al*. [1]. have argued for “programmes that improve secondary school environment and connectedness [because] they are the most promising large-scale interventions for improving health outcomes in adolescence” (p. 1647).

## Conclusions

1. Participation in the classroom and teacher and peer support were all determinants associated with positive health among both boys and girls. Because of the positive association with health in relation to peers, democratic practice and participation, it is important to develop opportunities for positive peer relations and increased democratic classroom participation.
2. There were differences related to less positive health and to a lower experience of participation and support from teachers and parents among girls in comparison with boys. Further research is needed to better understand the significance of sex and gender patterns in the school context.
3. The results could be helpful in understanding how health could be enabled and promoted from a salutogenic perspective in the school environment.
